# From Our Foreign Correspondent

**Published:** 1987-05

**Authors:** 


### From Our Foreign Correspondent

### A Remarkable Success Story in the Nile Delta

An invitation to visit Mansoura University to view their local progress and to give four lectures was accepted with enthusiasm and considerable interest, though with some reservations as my wife was reluctant to leave Bristol where two new granddaughters had recently arrived. The Department of Urology and Nephrology at Mansoura had already been recognised in Egypt as one of the leading centres for this specialty by the award eight years ago of one of the first gold medals of the Egyptian Urological Association to our colleague and former Registrar at Southmead Hospital, Professor Mohamed Ghoneim. It was Mohamed who had arranged the invitation and I fully anticipated finding a high standard of work. I was agreeably surprised to find a very high quality of equipment and other facilities despite the sad state of the economy in Egypt. In fact the Department provided such an oasis of excellence it is worthy of a special report.

The Urology and Nephrology Department occupies a new and architecturally designed building within the University campus, elegantly landscaped and situated adjacent to the main University Hospital so that although administered as an independent unit it is not isolated from other specialties, or from the Department of General Surgery and General Medicine. The Department consists of a teaching and administrative unit, a hospital of 120 beds, a residency and, adjacent to the Department, there are full research facilities occupying three floors with a large animal house on the ground floor. A suite of three operating theatres and an endoscopy room with twin tables is used to full capacity five days a week.

I was driven up an impressive ramp to the entrance of the teaching hospital, where I was received by Mohamed in a splendid foyer artistically designed around an essentially functional layout. The next floor was approached by a neat staircase curving round a small, well tended indoor garden. Mohamed's room was at the end of the corridor where it gave an immediate impression of efficient organisation and attention to detail as well as scrupulous cleanliness, yet at the same time a comfortable air of studious tranquility. From the window on two sides of the room there were attractive views across the University campus, where the gardens were planned around many old and interesting trees and lawns.

Already I heard the first of the effects of the Aswam high dam and the loss of the annual flood in Lower Egypt – namely compaction of the soil in the gardens which now required a system of drainage to avoid the plants standing with their roots in stagnant water. But the major medical effects were in the possible explanation of an increased incidence of transitional cell carcinoma of the bladder as compared with the incidence of squamous cell carcinoma induced by bilharzial infestation. The loss of the annual flood has reduced the amount of silt being washed down to the Nile Delta, consequently more artificial fertiliser now has to be used in the form of nitrates. This poses the question whether the nitrates in turn increase the amount of nitrosamines in the urine and hence the increased incidence of transitional cell carcinoma.

At the same time the small reduction of bilharzial infestation may be due to fewer snails being washed down the Nile, though it would be a happier thought that improved sanitation and better education of the peasant farmers was the factor responsible. However, there has been a corresponding increase in bilharzia in upper Egypt due to an increase in the snail population which used to be washed down the Nile at the time of the annual flood.

The research projects at present in progress included an interesting investigation into the possible production of a monoclonal antibody against keratin which is so prolific on the surface of a squamous cell carcinoma due to bilharzia. Their work in this field is already showing success and on this we had a long and interesting discussion about the future conjugation possibilities with any monoclonal antibody that may be prepared.

The hospital records were all transcribed onto computer, giving immediate access to the patients case history and notes, as well as extremely rapid retrieval of figures required for research statistics. Not only was this computer capable of registering the patient's name, hospital number and other personal details in arabic, but it could at the same time record the case histories in English. The computer has been constructed to recognise words rather than code numbers so that it would accept, for example, 'kidney' and 'renal' as identical, 'stone', 'lithiasis', 'calculus' as all three identical. If the side (left or right) had been omitted the computer would demand correction. The programming had been so carefully prepared that coding and decoding were not necessary and thereby eliminating one factor of possible human error. Like all computer programming the quality of the information retrieval depends on a high standard of programming as well as accurate transcribing from the handwritten notes and here the scribing had been streamlined as much as possible.

I was privileged to watch a renal transplantation from a live donor (harvesting a cadaver kidney would not be acceptable in a Moslem country). They have now completed 200 kidney transplants with a success rate favourably comparable to the results achieved in transplant centres in the West doing more than 50 per year. Two total cystectomies were observed with subsequent construction of a continence pouch in each case. Amongst other operations I was able to see two percutaneous endoscopic nephrolithotomies and three transurethral resections were observed via the dual viewing attachment. All had been thoroughly investigated beforehand and the meticulous quality of the surgical technique was a joy to watch.

Mohamed's immediate plans for the future are a C.T. scanner and an extracorporeal shock-wave lithotriptor which he hopes will be in position within the next twelve months. Nuclear magnetic resonance or Magnetic resonance imaging (M.R.I.) will probably be rather longer in the future.

My personal brief was to give four lectures, in two of which the subject was related to trauma of the urinary tract. To lecture on this subject in the Middle East and especially in Egypt means considering the problem of incidental urinary tract infection, which is rare amongst trauma casualties in the United Kingdom. In Egypt the incidence today of bilharzial infestation is 15 to 20%; of those a quarter to a third are secondarily infected, which presents an additional serious complication from extravasation of urine which may result in pelvic cellulitis and a gangrenous cutaneous cellulitis of suprapubic and perineal skin. (The lecture theatre was fully equipped with the standard audiovisual aids and furnished almost to the point of luxury.)

I was treated with the kindest consideration in every way by Mohamed Ghoneim. I fully anticipate that this Urology unit will soon acquire a world wide reputation for excellence.

J. P. Mitchell

